# Impact of Lamivudine-Based Antiretroviral Treatment on Hepatitis B Viremia in HIV-Coinfected South Africans

**DOI:** 10.3390/v12060634

**Published:** 2020-06-11

**Authors:** Azwidowi Lukhwareni, Maemu Petronella Gededzha, Edina Amponsah-Dacosta, Jason T. Blackard, Rosemary J. Burnett, Selokela Gloria Selabe, Thanda Kyaw, M. Jeffrey Mphahlele

**Affiliations:** 1 HIV and Hepatitis Research Unit, Department of Virology, Sefako Makgatho Health Sciences University and National Health Laboratory Service, MEDUNSA, Pretoria 0204, South Africa; maemu.gededzha@nhls.ac.za (M.P.G.); eddiedacosta@yahoo.com (E.A.-D.); jason.blackard@uc.edu (J.T.B.); rose.burnett@smu.ac.za (R.J.B.); selokela.selabe@smu.ac.za (S.G.S.); thanda.k@gmail.com (T.K.); Jeffrey.Mphahlele@mrc.ac.za (M.J.M.); 2 National Health Laboratory Service, Tshwane Academic Division, Department of Medical Virology, University of Pretoria, Pretoria 0002, South Africa; 3 Department of Molecular Medicine and Haematology, Faculty of Health Sciences, University of Witwatersrand and National Health Laboratory Service, Johannesburg 2193, South Africa; 4 Vaccines for Africa Initiative, School of Public Health and Family Medicine, University of Cape Town, Cape Town 7700, South Africa; 5 Division of Digestive Diseases, Department of Internal Medicine, University of Cincinnati College of Medicine, Cincinnati, OH 45267, USA; 6 South African Medical Research Council, Soutpansberg Road, Pretoria 0118, South Africa

**Keywords:** lamivudine, antiretroviral therapy (ART), hepatitis B virus (HBV), HIV coinfection, South Africa

## Abstract

This prospective study investigated the impact of lamivudine-containing antiretroviral therapy (ART) on HIV-positive patients in South Africa with baseline hepatitis B virus (HBV) infection. Follow-up samples from 56 HBV/HIV co-infected patients, 25 with occult HBV infection (OBI) and 31 with chronic HBV infection (CHB), were available for analysis. HBV viral loads were quantified at 6, 12, 18, and 24 months post-ART initiation by the COBAS TaqMan HBV Test 48 assay, and the HBV polymerase gene was amplified with an in-house nested polymerase chain reaction assay. During 24 months of lamivudine-based ART, 6 of 8 (75%) OBI and 4 of 6 (67%) CHB patients achieved undetectable levels of HBV DNA, while 2 patients had persistent HBV DNA levels ≥ 2 × 10^5^ despite lamivudine-based ART for 24 months. HIV viremia was undetectable in all patients at 12 months, suggesting high adherence to ART. Several lamivudine-associated HBV resistance mutations, including L180M, A181T, M204I, and M204V, were observed. Sequence analysis also revealed a rare genotype G infection. While resource-limited settings may use lamivudine-based ART because of availability and low cost, antivirals with dual therapy against HBV and HIV (e.g., lamivudine and tenofovir) should always be recommended with the regular monitoring of HBV viremia levels.

## 1. Introduction

Human-immunodeficiency-virus (HIV) infection alters the course of the hepatitis B virus (HBV) disease by increasing the rate of chronicity, enhancing HBV replication, and accelerating liver-related morbidity and mortality [1,2,3,4,5,6,7]. In addition, HIV complicates the diagnosis of HBV infection by generating atypical serological presentations, such as occult HBV infection (OBI), defined as the detection of viral DNA in the blood in the absence of hepatitis B surface antigen (HBsAg) [2,8,9]. HBsAg prevalence in HIV-positive South African pregnant women ranges from 3.4% to 6.2% [10,11]. However, HBV prevalence in HIV-positive patients who have developed acquired immunodeficiency syndrome (AIDS) tends to be higher, with a study on South Africans initiating antiretroviral therapy (ART) reporting HBsAg, and HBV DNA prevalence of 22.9% and 40.6%, respectively [4].

South Africa is among the first African countries to formally adopt the World Health Organization’s (WHO) Universal Test and Treat (UTT) guidelines on HIV treatment in support of the goals of the Joint United Nations Program on HIV/AIDS (UNAIDS) that 90% of people living with HIV are aware of their status, 90% of people with diagnosed infection receive ART, and 90% of individuals receiving ART achieve viral suppression. While expanding HIV treatment programs, significant challenges remain when managing HBV/HIV coinfected patients [7,12,13]. The current Southern African ART guidelines recommend testing for HBsAg before initiating ART to ensure that HIV/HBV-coinfected patients are placed on an appropriate ART regimen containing drugs that are also active against HBV, such as tenofovir and lamivudine (or emtricitabine) [7]. Importantly, given the high prevalence of OBI in HIV-positive patients, a large proportion of HIV/HBV coinfections are not identified by HBsAg screening alone. Lamivudine is commonly associated with HBV resistance mutations, whereas tenofovir is not [14]. In addition, patients with seronegative OBI (i.e., negative for all serological markers) may maintain persistent low-level HBV viremia during long-term lamivudine-containing ART, resulting in the reactivation of chronic HBV infection (CHB) and hepatic enzyme flares [9]. Furthermore, after initiation of lamivudine-containing ART, patients with overt CHB may progress to persistent OBI [9], which may be misinterpreted as HBV suppression if follow-up testing only consists of HBsAg. Although the cost of tenofovir is significantly declining, it remains relatively expensive compared to lamivudine, and access to tenofovir is limited in many African countries.

At the time of this study, HIV-positive patients initiating ART were placed on a first-line regimen consisting of lamivudine, stavudine, and efavirenz without being screened for HBsAg. This study investigated the impact of a lamivudine-containing ART regimen on HBV (both CHB and OBI) during management of HIV/AIDS patients in Pretoria, South Africa.

## 2. Materials and Methods

### 2.1. Study Design and Study Population

This prospective study included plasma samples collected between August 2004 and March 2007 (stored at −70 °C) [4] from patients initiating lamivudine-containing ART (i.e., lamivudine, stavudine, and efavirenz) as part of a state hospital HIV/AIDS management program conducted at the Tshepang HIV outpatient clinic of the Dr George Mukhari Academic Hospital in Pretoria. Of 78 participants in the parent study, current analysis was confined to HBV DNA-positive plasma samples from 56 HIV/AIDS patients, consisting of 32 females and 24 males with a mean age of 35 years (range: 20–51) who had been longitudinally followed for 6–24 months (Table 1).

### 2.2. Ethics Approval

The study was approved by the Medunsa Research, Ethics, and Publications Committee of the Faculty of Medicine at the University of Limpopo, Medunsa Campus (now Sefako Makgatho Health Sciences University), and the Dr George Mukhari Academic Hospital Ethics Committee (Ethics number: MP 07/2005).

### 2.3. HBV Serological and Liver Function Testing

All patients had pre-ART initiation (baseline) plasma samples that were screened for HBsAg, antibodies to the HBV core antigen (anti-HBc), and antibodies to HBsAg (anti-HBs) (Elecsys ^®^ 2010 Immunoassay Analyzer, Roche Diagnostics, Penzberg, Germany) as previously reported [4]. At the baseline, 31 individuals were HBsAg-positive (i.e., CHB), while 25 were HBsAg-negative (i.e., OBI). Follow-up samples were evaluated for HBsAg when available. Mean baseline alanine aminotransferase (ALT) was 33.9 IU/L (range: 8–304; Table 1). All baseline samples were seronegative for hepatitis C virus using the Axsym 3.0 assay (Abbott, Chicago, IL, USA).

### 2.4. HBV DNA Detection

Viral nucleic acid was extracted from 200 μL of serum with the High Pure Viral Nucleic Acid kit (Roche Diagnostics, Penzberg, Germany), eluted in 50 μL, and used immediately or stored at −70 °C for later use. The HBV polymerase gene was amplified by nested polymerase chain reaction (PCR) using two sets of primers that flank the YMDD motif of the HBV polymerase gene [4,15,16]. The first round primers were 5’–GTC TGC GGC GTT TTA TCA–3’ (nucleotide positions 381–398 according to *Eco RI* restriction site), and 5’–GGA GTT CCG CAG TAT GGA TCG G–3’ (positions 1282–1261) that target a 902 base pair (bp) product. Second-round primers were 5’–GGT ATG TTG CCC GTT TGT CC–3’ (positions 458–477) and reverse 5’–GGC GAG AAA GTG AAA GCC T–3’ (positions 1103–1085) that amplify a 646 bp product. The lower detection limit of the assay was 1.0 IU/mL (5.6 copies/mL) [4,15,16].

### 2.5. HBV DNA Quantification

Baseline samples were evaluated for HBV DNA as part of a previous study [4]. HBV DNA levels were quantified in follow-up samples (i.e., 6, 12, 18, and 24 months) using the COBAS TaqMan HBV Test 48 assay (Roche Diagnostics, Penzberg, Germany) [17]. Samples were considered detectable when DNA levels were ≥ 6.00 IU/mL, and undetectable when < 6.00 IU/mL. 

### 2.6. Sequence Analysis

HBV PCR products were sequenced directly using the Spectru-Medix SCE 2410 Genetic Analysis System (Spectru-Medix LLC, State College, PA, USA). Evolutionary analysis was executed using Bayesian Evolutionary Analysis by Sampling Trees v1.8.4 [18] with an uncorrelated log-normal relaxed molecular clock, generalized time-reversible model, and nucleotide-site heterogeneity with gamma distribution. The analysis was run with a chain length of 100,000,000. Effective sample sizes were >1000, indicating sufficient sampling. A maximal clade credibility tree was selected after a 10% burn-in by TreeAnnotator v1.8.4. Nucleotide sequences were submitted to GenBank under accession numbers MH431627–MH431693. Sequences were also evaluated for HBV drug-resistance mutations and quality control using an online tool available in the rtHBV DB database through Stanford University.

### 2.7. HIV Viral Load and CD4+ Cell Counts

HIV viral loads were quantified during routine HIV management by the Virology Diagnostic Laboratory, National Health Laboratory Service, using the NucliSens EasyQ HIV-1 assay (BioMe’rieux, Boxtel, Netherlands). CD4+ cell counts were also determined as part of routine HIV treatment by an independent team from the Haematology Diagnostic Laboratory, National Health Laboratory Service using the Beckman Coulter MPL/CellMek fully automated flow-cytometer system (Beckman Coulter, Fullerton, CA, USA).

## 3. Results

### 3.1. Baseline Characteristics

At baseline, 33.9% of subjects (19/56) had ≥ 2000 IU/mL HBV DNA, with 36.8% (7/19) having HBV DNA ≥ 20,000 IU/mL (Table 2). Of the OBI baseline samples, HBV DNA was undetectable in 12% (3/25), and 8% (4/25) had ≥ 2000 IU/mL HBV DNA with 50% (2/4) having ≥ 20,000 IU/mL (Table 2). Despite some samples having no quantifiable HBV DNA with the COBAS TaqMan HBV Test 48 assay, they were considered OBI because they were HBV DNA-positive, indicated by an in-house qualitative PCR assay.

**Figure 1:**
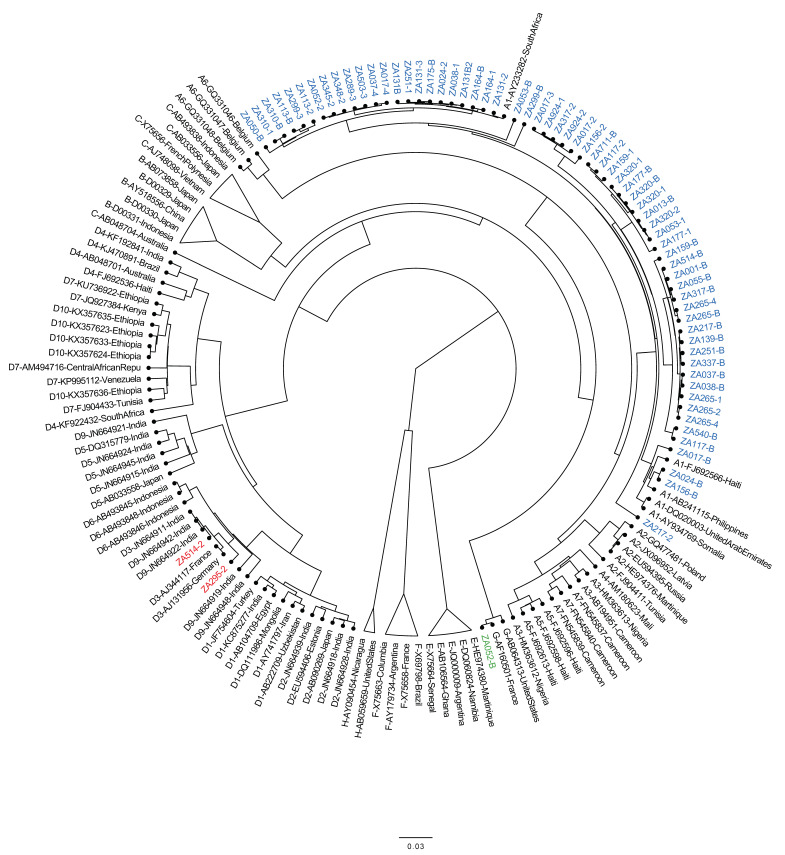
Phylogenetic analysis of HBV polymerase open reading from South Africans with occult or chronic HBV. Sequences from this study are indicted by “ZA”, while references are indicated by subgenotype–accession number–country. Genotype A sequences from this study are highlighted in blue, genotype D in red, and genotype G in green.

Of the CHB baseline samples, HBV DNA was undetectable in 6.5% (2/31), and 61.3% (19/31) had ≥ 2000 IU/mL HBV DNA, with 73.7% (14/19) having ≥ 20,000 IU/mL (Table 2). Baseline HIV viral-load results were available for 41 patients, with 95.1% (39/41) having ≥ 1000 copies/mL (Table 2). Baseline CD4+ results were available for 47 patients, with 97.9% (46/47) having ≤ 350 cells/mL (Table 2). As shown in Figure 1, HBV genotypes were determined for 30 individuals and included A (*n* = 27), D (*n* = 2), and G (*n* = 1).

### 3.2. Clinical Responses during Six Months of Lamivudine-Based ART

Samples for HBV DNA quantification at the six-month follow-up were available for 36 patients. HBV DNA was detectable in 55.56% (20/36), with 25% (5/20) having ≥ 2000 IU/mL (Table 2). HBV DNA was undetectable in 50% (9/18) of patients with OBI at baseline versus 38.9% (7/18) of patients with CHB at baseline. HBV DNA levels ≥ 2 × 10^5^ IU/mL were found only in 11.1% (2/18) of patients with baseline CHB (Table 2). Of the 36 individuals, 21 were tested for HBsAg, and 6 of 21 (28.6%) were HBsAg-positive. Two of these patients had OBI at baseline. Of the remaining 71.4% (15/21) testing HBsAg negative at 6 months, 4 were HBsAg positive at baseline.

HIV viral loads were available for 22 patients, distributed as follows: <50 copies/mL, 81.1% (18/22); < 1000 copies/mL, 13.6% (3/22); ≥ 1000 copies/mL, 4.6% (1/22) (Table 2). CD4+ cell counts were available for 24 patients and distributed as follows: < 50 cells/mL, 8.3% (2/24), 50 to ≤ 350 cells/mL, 75% (18/24), and > 350 cells/mL, 16.7% (4/24) (Table 2).

### 3.3. Clinical Responses during 12 Months of Lamivudine-Based ART

At the 12-month follow-up visit, 34 samples were available for HBV DNA testing. HBV DNA was undetectable in 50% (17/34), with levels in the remaining 50% ranging from < 10 to ≥ 2 × 10^5^ IU/mL (Table 2). Of these, 23.5% (4/17) (samples ZA265, ZA320, ZA131, and ZA024) had HBV DNA ≥ 20,000 IU/mL. Three of these had baseline viremia of > 2 × 10^5^ IU/mL, while the fourth was undetectable but HBsAg-positive at baseline (Table 3). Of the 34 patients, 20 (58.8%) had HBV DNA results at baseline, 6, and 12 months as follows: 30% (6/20) undetectable; 20% (4/20) < 10 IU/mL; 25% (5/20) 10 to ≤ 2000 IU/mL; 5% (1/20) ≥ 2000 to < IU/mL; 5% (1/20) ≥ to < 2 × 10^5^ IU/mL; and 15% (3/20) ≥ 2 × 10^5^ IU/mL. The profiles of the 20 patients are shown in Table 3. Two of the three patients with HBV DNA ≥ 2 × 10^5^ IU/mL were HBsAg-positive at 6 and 12 months, while the third patient was not tested for HBsAg at 12 months due to an insufficient sample, but was HBsAg positive at the six-month follow-up.

All 19 specimens tested for HIV viremia had undetectable levels of < 50 copies/mL. CD4+ cell counts were available for 11 patients, with 63.6% (7/11) having between 50 and ≤ 350 cells/mL and 36.4% (4/11) having > 350 cells/mL (Table 2).

### 3.4. Clinical Responses during 18–24 Months of Lamivudine-Based ART

At the 18-month follow-up visit, 26 samples were available for HBV DNA testing. Of these, 46% (12/26) had detectable viremia (Table 2) and 30.8% (8/26) had follow-up serial bleeds at baseline, six, 12 and 18 months (Table 4). HBV DNA > 20,000 IU/mL was detected in 37.5% (3/8) (samples ZA265, ZA320, and ZA113). Two patients had CHB at baseline with baseline viremia of > 20,000 IU/mL (Table 4).

At the 24 months follow-up visit, 14 samples were available for HBV DNA testing. Of these, four (28.6%) had detectable viremia with 50% (2/4) (samples ZA320 and ZA265) having viremia of > 2 × 10^5^ IU/mL. At baseline, sample ZA265 was CHB and sample ZA320 was OBI with >1.1 × 10^8^ IU/mL HBV DNA (Table 5).

### 3.5. HBV Treatment Failure, Drug-Resistance Mutations, and Genotyping

Of the patients followed up at 6, 12, 18, and 24 months, a total of 6 patients had not suppressed HBV replication. At their last follow-up visit, 5 patients had HBV DNA > 2 × 10^5^ IU/mL, while one patient had HBV DNA > 2 × 10^3^ IU/mL. Analysis of polymerase gene sequences from baseline and follow-up samples showed the presence of resistance-associated mutations in 5 of these 6 patients, including L180M, A181T, M204I, and M204V (Table 5). Patient ZA113 had the L180M mutation and the L180M + M204V mutations at the 12- and 18-month follow-up visits, respectively, with HBV viremia increasing from 1.99 × 10^4^ IU/mL at baseline to 7.08 × 10^6^ at the 18-month follow-up. At baseline, patient ZA164 was HBsAg-positive with 7.67 × 10^3^ IU/mL HBV DNA and the M204I variant. At the final visit at 6 months, HBV DNA level had dropped to 2.42 × 10^3^ IU/mL, the M204I variant was not detectable, and the patient was still HBsAg-positive. Patient ZA131 was HBsAg-positive with undetectable HBV DNA levels at baseline, which increased to 3.39 × 10^2^ IU/mL at 6 months with the M204I variant being detected. At this patient’s final follow-up at 12 months, HBV viral load had increased considerably to 1.1 × 10^8^ IU/mL, but there were no mutations detected, and the patient was still HBsAg-positive (Table 5).

## 4. Discussion

Monitoring HBV viremia during treatment is the standard of care for the timely detection of drug resistance [7,12,19,20].This study investigated the impact of lamivudine-based ART regimens on HBV viremia for the management of HIV/AIDS in South African patients in whom HIV and HBV prevalence rates are high. Several guidelines have been developed for the treatment of CHB in HBV/HIV coinfected patients, although the optimal timing of treatment initiation is still debated [19,20]. HBV viral load is a strong predictor for the development of hepatocellular carcinoma and is important in treatment success [13,19,20,21].

The current study demonstrated that patients on ART suppressed HBV DNA to undetectable levels. At 6 months, 44.4% had viral suppression, and 50% at 12 months. The benefit of lamivudine-based ART regimens was also demonstrated at 18 and 24 months: 53.9% and 71.4% had undetectable HBV viremia, respectively. A study from China demonstrated that over 50% of HBV/HIV-coinfected patients with HBV DNA of < 20,000 IU/mL were able to achieve HBV suppression when treated either with lamivudine monotherapy or dual tenofovir and lamivudine therapy [22]. Regardless of baseline HBV DNA levels, the results of the current study are supported by other HBV/HIV coinfection studies demonstrating that 30–60% of patients achieved HBV DNA suppression with lamivudine monotherapy at 48 weeks [22,23,24,25,26].

In the current study, patients who experienced persistent HBV viremia with viral loads > 20,000 IU/mL, even after 18 and 24 months on a lamivudine-based HAART regimen, had baseline HBV DNA levels ≥ 20,000 IU/mL. Thus, high HBV baseline viremia may explain the lack of viral suppression in the absence of HBV drug-resistance-associated variants among these patients. A multinational HBV/HIV-coinfection study compared lamivudine- or emtricitabine-based monotherapy, and HBV dual therapy with tenofovir disoproxil fumarate and lamivudine or emtricitabine, and found that HBV/HIV-coinfected patients with baseline HBV DNA of < 20,000 IU/mL responded well regardless of treatment regimen. In addition, subjects in the monotherapy group who did not maintain a treatment response over time had HBV DNA ≥ 20,000 IU/mL [27]. Other studies from resource-limited settings demonstrated that HBV/HIV-coinfected patients with HBeAg-negative disease were likely to have low levels of HBV DNA, while patients with HBV DNA of < 20,000 IU/mL on lamivudine-based treatment could maintain viral suppression for nearly three years [22,23,25,27,28].

Mutational analysis for the current study found patients infected with lamivudine-resistant variants. Previous studies from South Africa reported the detection of HBV-resistance mutations associated with lamivudine in treatment-naïve and -experienced individuals with CHB and OBI [16,29]. The current study also detected the M204I variant at baseline in treatment-naïve patients, and other mutations such as A181T, L180M, or M204V during follow-up of individuals on a lamivudine-based regimen. All samples with resistance mutations had HBV viremia ≥ 7.67 × 10^3^ IU/mL (Table 5). Previous studies reported the risk of developing resistance to be associated with baseline HBV DNA levels ≥ 20,000 IU/mL, especially in patients on lamivudine monotherapy, and a slow decline in HBV DNA level [20,27]. A recent study from China with a small number of HBV/HIV-coinfected patients demonstrated that lamivudine monotherapy was associated with higher rates of lamivudine-resistant HBV compared to tenofovir and lamivudine dual therapy, but did not stratify their analyses by baseline HBV DNA levels [26]. While phylogenetic analysis demonstrated the predominant HBV genotypes to be A and D in agreement with other reports from South Africa [30,31], genotype G was also detected for the first time in South Africa.

Limitations of the study include the modest sample size that was affected by attrition and missing data at some time points. In addition, the full profile of HBV serological markers could not be performed on every sample due to insufficient sample volumes. Adherence to therapy could not be confirmed, although HIV viremia was undetectable in all patients at 12 months suggesting high adherence. Nonetheless, the need to screen HIV-infected patients for HBV coinfection prior to ART initiation as part of the UTT strategy cannot be overemphasized. Finally, while some samples taken at 18 and 24 months had no resistance-associated mutations, routine population-based Sanger sequencing was conducted for this study that did not allow for the detection of minor variant populations. Thus, the magnitude of drug resistance may have been underestimated.

Many authors discourage using lamivudine as the only anti-HBV drug within an ART regimen among patients with high HBV DNA levels due to its low potency and the high risk for mutations that confer cross-resistance to other anti-HBV drugs such as emtricitabine, telbivudine, and entecavir [19,27]. However, lamivudine-based monotherapy may be considered in patients with low HBV DNA levels, as typically occurs during occult HBV infection when other HBV active drugs are not available. Others also called for strategic use of lamivudine-based therapies due to their low cost and availability in resource-limited settings [32,33]. In general, the use of lamivudine as the only anti-HBV drug within an ART regimen is strongly discouraged for HBV/HIV-coinfected individuals, but may be considered for individuals with low HBV DNA levels, as typically occurs during occult HBV infection and in the absence of liver cirrhosis.

## Figures and Tables

**Table 1:** Baseline characteristics of patients before lamivudine-based ART treatment.

Characteristics	HBsAg Positive (*n* = 31)	HBsAg Negative (*n* = 25)
Mean age (range), years	35.5 (20–51)	35.6 (24–50)
Male/female	13/18	11/14
Mean HBV DNA (range), IU/mL	7.1 × 10^6^ (UND–> 1.10 × 10^8^)	4.5 × 10^6^ (UND–1.10 × 10^8^)
Mean CD4+ count (range), cells/mm^3^	114 (5–1069)	101.6 (2–241)
Mean HIV RNA (range), copies/mL	217,950 (50–750,000)	148,405 (200–650,224)
Mean ALT (range), U/L	30.5 (8–187)	34.3 (10–304)

UND–Undetectable viral load.

**Table 2:** Clinical characteristics during treatment with lamivudine-based ART.

**Characteristics**	**Time Post-ART Initiation**				
	Baseline	6 Months	12 Months	18 Months	24 Months
**HBV DNA (IU/mL)—occult and chronic**	***n* = 56 (100%)**	***n* = 36 (100%)**	***n* = 34 (100%)**	***n* = 26 (100%)**	***n* = 14 (100%)**
Undetectable	5 (8.93)	16 (44.44)	17 (50.00)	14 (53.8)	10 (71.43)
<10	6 (10.71)	8 (22.22)	6 (17.70)	4 (15.4)	1 (7.14)
10 to <2000	26 (46.43)	7 (19.45)	6 (17.70)	4 (15.4)	1 (7.14)
≥ 2000 to <20,000	12 (21.43)	3 (8.33)	1 (2.90)	1 (3.9)	0
≥ 20,000 to < 2 × 10^5^	3 (5.36)	0	1 (2.90)	1 (3.9)	0
≥ 2 × 10^5^	4 (7.14)	2 (5.56)	3 (8.80)	2 (7.6)	2 (14.29)
**HBV DNA (IU/mL)–occult**	***n* = 25 (100%)**	***n* = 18 (100%)**	***n* = 16 (100%)**	***n* = 13 (100%)**	***n* = 8 (100%)**
Undetectable	3 (12)	9 (50)	6 (38)	7 (53.8)	6 (75)
<10	4 (16)	5 (27.7)	5 (31)	1 (7.7)	1 (12.5)
10 to <2000	14 (56)	3 (16.7)	4 (25)	4 (30.8)	0
≥ 2000 to 20,000	2 (8)	1 (5.6)	0	0	0
≥ 20,000 to <2 × 10^5^	0	0	0	0	0
≥ 2 × 10^5^	2 (8)	0	1 (6)	1 (7.7)	1 (12.5)
**HBV DNA (IU/mL)—chronic**	***n* = 31(100%)**	***n* = 18 (100%)**	***n* = 18 (100%)**	***n* = 13 (100%)**	***n* = 6 (100%)**
Undetectable	2 (6.45)	7 (38.9)	11 (61)	7 (53.9)	4 (66.6)
<10	3 (9.7)	3 (16.7)	1 (5.6)	3 (23)	0
10 to <2000	12 (38.7)	4 (22.2)	2 (11.1)	0	1 (16.7)
≥ 2000 to 20,000	9 (29.0)	2 (11.1)	1 (5.6)	1 (7.7)	0
≥ 20,000 to <2 × 10^5^	3 (9.7)	0	1 (5.6)	1 (7.7)	0
≥2 × 10^5^	2 (6.45)	2 (11.1)	2 (11.1)	1 (7.7)	1 (16.7)
**CD4+ count (cells/mm^3^)**	***n* = 47(100%)**	***n* = 24(100%)**	***n* = 11(100%)**		
<50	20 (42.6)	2 (8.3)	0		
50 to ≤ 350	26 (55.3)	18(75)	7 (63.6)		
>**350**	1 (2.1)	4 (16.7)	4 (36.4)		
**Plasma HIV RNA (copies/mL)**	***n* = 41**	***n* = 22**	***n* = 19**		
Undetectable (<50)	0	18(81.8)	19(100)		
<1000	2 (4.9)	3 (13.6)	0		
≥ 1000	19 (46.3)	1 (4.6)	0		
>10^5^	20 (48.8)	0	0		

**Table 3:** HBV virologic response to lamivudine-based ART over 12 months of follow-up.

ID	HIV RNA				Baseline				6-month follow-up			12-month follow-up		
	Baseline	Month 6	Month 12	HBsAg		Anti-HBs	Anti-HBc	HBV DNA	HBV DNA	HBsAg	Mutation	HBV DNA	HBsAg	Mutation
ZA053		ND	ND	−		+	−	TND	7.45 × 10^1^	−		<6.00 × 10^0^	−	
ZA265	410,000	<50	<50	+		−	-	>1.10 × 10^8^	7.36 × 10^2^	+		1.52 × 10^5^	+	
ZA320	171,533	<50	<50	−		−	+	>1.10 × 10^8^	1.05 × 10^4^	+	A181T	>1.10 × 10^8^	ND	
ZA345	62,000	<50	<50	−		−	−	1.58 × 10^2^	<6.00 × 10^0^	−		7.5 × 10^0^	−	
ZA113	750,000	147	<50	+		−	+	1.99 × 10^4^	2.23 × 10^5^	ND		8.76 × 10^3^	ND	L180M
ZA156	29,000	<50	<50	−		+	−	TND	TND	−		4.09 × 10^2^	−	
ZA117	ND	<25	55	+		−	−	7.94 × 10^4^	7.5 × 10^0^	−		5.73 × 10^2^	−	
ZA177	23,000	ND	ND	−		−	+	3.61 × 10^1^	<6.00 × 10^0^	+		9.68 × 10^0^	ND	
ZA024	51,000	74,784	ND	−		+	+	4.46 × 10^3^	<6.00 × 10^0^	−		>6.00 × 10^0^	−	
ZA013	97,000	<50	<50	−		+	+	2.95 × 10^2^	TND	−		TND	−	
ZA467	ND	ND	ND	+		−	−	3.10 × 10^1^	<6.00 × 10^0^	ND		TND	ND	
ZA131	642,499	<50	<50	+		−	−	TND	3.39 × 10^2^	+	M204I	>1.10 × 10^8^	+	
ZA047	97,000	<50	<50	−		−	−	TND	1.10 × 10^3^	−		TND	−	
ZA217	ND	ND	ND	−		−	+	1.14 × 10^6^	TND	ND		1.10 × 10^1^	ND	
ZA540	ND	220	<50	−		+	+	2.40 × 10^2^	TND	−		TND	ND	
ZA251	25,000	<50	<50	+		−	−	3.40 × 10^2^	<6.00 × 10^0^	−		TND	ND	
ZA212	ND	ND	ND	+		−	−	TND	TND	ND		TND	ND	
ZA024	438,585	ND	ND	+		−	+	>1.10 × 10^8^	7.58 × 10^3^	ND	M204I	>1.10 × 10^8^	ND	
ZA064	213,140	<50	<50	−		−	+	<6.00 × 10^0^	TND	ND		3.45 × 10^1^	ND	
ZA348	ND	ND	ND	+		−	−	2.74 × 10^1^	TND	ND		2.81 × 10^1^	ND	

ND = not done; TND = target not detected.

**Table 4:** HBV virologic response to lamivudine-based ART over 18 months of follow-up.

ID	HIV RNA			Baseline				HBV DNA								
	Baseline	Month 6	Month 12	HBsAg	Anti-HBs	Anti-HBc	HBV DNA	6-Month Follow-Up			12-Month Follow-Up			18-Month Follow-Up		
								HBV DNA	HBsAg	Mutation	HBV DNA	HBsAg	Mutation		HBV DNA	Mutation
ZA265	410,000	< 50	< 50	+	−	−	> 1.10 × 10^8^	7.36 × 10^2^	+		1.52 × 10^5^	+		2.49 × 10^4^		
ZA320	171,533	< 50	< 50	-	−	+	> 1.10 × 10^8^	1.05 × 10^4^	+	A181T	> 1.10 × 10^8^	ND		3.48 × 10^7^		
ZA177	23,000	ND	ND	−	−	+	3.61 × 10^1^	< 6.00 × 10^0^	+		9.68 × 10^0^	ND		TND		
ZA024	51,000	74,784	ND	−	+	+	4.46 × 10^3^	< 6.00 × 10^0^	−		> 6.00 × 10^0^	−		5.85 × 10^1^		
ZA013	97,000	< 50	< 50	−	+	+	2.95 × 10^2^	TND	−		TND	−		7.91 × 10^0^		
ZA467	ND	ND	ND	+	−	−	3.10 × 10	< 6.00 × 10^0^	ND		TND	ND		TND		
ZA117	ND	< 25	55	+	−	−	7.94 × 10^4^	7.5 × 10^0^	−		5.73 × 10^2^	−		TND		
ZA113	750,000	147	< 50	+	−	+	1.99 × 10^4^	2.23 × 10^5^	ND		8.76 × 10^3^	ND	L180M	7.08 × 10^6^		L180MM204V

ND = not done; TND = target not detected.

**Table 5:** Profile of patients with/without mutations and persistent HBV DNA from baseline to 24 months.

ID	HIV viral load			Baseline HBV					HBV DNA									
	Baseline	Month 6	Month 12	HBsAg	Anti-HBs	Anti-HBc	HBV DNA	Mutation	6-month follow-up			12-month follow-up			18-month follow-up		24-month follow-up	
									HBV DNA	HBsAg	Mutation	HBV DNA	HBsAg	Mutation	HBV DNA	Mutation	HBV DNA	Mutation
ZA265	410,000	< 50	< 50	+	−	−	> 1.10 × 10^8^	−	7.36 × 10^2^	+	−	1.52 × 10^5^	+	−	2.49 × 10^4^	−	2.53 × 10^5^	None
ZA320	171,533	< 50	< 50	-	−	+	> 1.10 × 10^8^	−	1.05 × 10^4^	−	A181T	> 1.10 × 10^8^	ND	−	3.48 × 10^7^	−	1.14 × 10^6^	None
ZA113	750,000	147	< 50	+	−	+	1.99 × 10^4^	−	2.23 × 10^5^	ND	−	8.76 × 10^3^	ND	L180M	7.08 × 10^6^	L180MM204V		
ZA024	438,585	ND	ND	+	−	+	> 1.10 × 10^8^	−	7.58 × 10^3^	ND	M204I	> 1.10 × 10^8^	ND	−				
ZA131	642,499	< 50	< 50	+	−	−	TND	−	3.39 × 10^2^	+	M204I	> 1.10 × 10^8^	+	−				
ZA164	64,000	< 50	< 50	+	−	+	7.67 × 10^3^	M204I	2.42 × 10^3^	+	−							

ND = not done; TND = target not detected.

## References

[1] Benhamou Y., Bochet M., Di Martino V., Charlotte F., Azria F., Coutellier A., Vidaud M., Bricaire F., Opolon P., Katlama C. (1999). Liver fibrosis progression in human immunodeficiency virus and hepatitis C virus coinfected patients. The Multivirc Group. Hepatology.

[2] Mphahlele M.J., Lukhwareni A., Burnett R.J., Moropeng L.M., Ngobeni J. (2006). High risk of occult hepatitis B virus infection in HIV-positive patients from South Africa. J. Clin. Virol..

[3] Hoffmann C.J., Charalambous S., Thio C.L., Martin D.J., Pemba L., Fielding K.L., Churchyard G.J., Chaisson R.E., Grant A. (2007). Hepatotoxicity in an African antiretroviral therapy cohort: The effect of tuberculosis and hepatitis B. AIDS.

[4] Lukhwareni A., Burnett R.J., Selabe S.G., Mzileni M.O., Mphahlele M. (2009). Increased detection of HBV DNA in HBsAg-positive and HBsAg-negative South African HIV/AIDS patients enrolling for highly active antiretroviral therapy at a Tertiary Hospital. J. Med Virol..

[5] Matthews P.C., Geretti A.M., Goulder P.J., Klenerman P. (2014). Epidemiology and impact of HIV coinfection with hepatitis B and hepatitis C viruses in Sub-Saharan Africa. J. Clin. Virol..

[6] Spearman C.W., Afihene M., Ally R., Apica B., Awuku Y., Cunha L., Dusheiko G., Gogela N., Kassianides C., Kew M. (2017). Hepatitis B in sub-Saharan Africa: Strategies to achieve the 2030 elimination targets. Lancet Gastroenterol. Hepatol..

[7] Meintjes G., Moorhouse M.A., Carmona S., Davies N., Dlamini S., van Vuuren C., Manzini T., Mathe M., Moosa Y., Nash J. (2017). Adult antiretroviral therapy guidelines 2017. South Afr. J. Hiv. Med..

[8] Firnhaber C., Viana R., Reyneke A., Schultze D., Malope B., Maskew M., Di Bisceglie A., MacPhail P., Sanne I., Kew M. (2009). Occult hepatitis B virus infection in patients with isolated core antibody and HIV co-infection in an urban clinic in Johannesburg, South Africa. Int. J. Infect. Dis..

[9] Amponsah-Dacosta E., Selabe S.G., Mphahlele M.J. (2018). Evolution of the serologic and virologic course of occult HBV infection in therapy experienced HIV co-infected patients. J. Med. Virol..

[10] Andersson M.I., Maponga T.G., Ijaz S., Barnes J., Theron G.B., Meredith S.A., Preiser W., Tedder R.S. (2013). The epidemiology of hepatitis B virus infection in HIV-infected and HIV-uninfected pregnant women in the Western Cape, South Africa. Vaccine.

[11] Burnett R.J., Ngobeni J.M., François G., Hoosen A.A., Leroux-Roels G., Meheus A., Mphahlele M. (2007). Increased exposure to hepatitis B virus infection in HIV-positive South African antenatal women. Int. J. Std. Aids..

[12] Nwokediuko S.C. (2011). Chronic hepatitis B: Management challenges in resource-poor countries. Hepat Mon..

[13] Spearman C.W., Sonderup M.W., Botha J.F., Van der Merwe S.W., Song E., Kassianides C., Newton K.A., Hairwadzi H. (2013). South African guideline for the management of chronic hepatitis B: 2013. South. Afr. Med. J..

[14] Hamers R.L., Zaaijer H.L., Wallis C.L., Siwale M., Ive P., Botes M.E., Sigaloff K.C., Hoepelman A.I., Stevens W.S., Tobias F. R.d.W. (2013). HIV-HBV coinfection in southern Africa and the effect of lamivudine- versus tenofovir-containing cART on HBV outcomes. J. Acquir. Immune Defic. Syndr..

[15] Gutfreund K.S., Williams M., George R., Bain V.G., Ma M.M., Yoshida E.M., Villeneuve J.P., Fischer K.P., Tyrrel D.L. (2000). Genotypic succession of mutations of the hepatitis B virus polymerase associated with lamivudine resistance. J. Hepatol..

[16] Selabe S.G., Lukhwareni A., Song E., Leeuw Y.G., Burnett R.J., Mphahlele M. (2007). Mutations associated with lamivudine-resistance in therapy-naïve hepatitis B virus (HBV) infected patients with and without HIV co-infection: Implications for antiretroviral therapy in HBV and HIV co-infected South African patients. J. Med. Virol..

[17] Wald O., Pappo O., Safadi R., Dagan-Berger M., Beider K., Wald H., Franitza S., Weiss I., Avniel S., Boaz P. (2004). Involvement of the CXCL12/CXCR4 pathway in the advanced liver disease that is associated with hepatitis C virus or hepatitis B virus. Eur. J. Immunol..

[18] Drummond A.J., Suchard M.A., Xie D., Rambaut A. (2012). Bayesian phylogenetics with BEAUti and the BEAST 1.7. Mol. Biol. Evol..

[19] Terrault N.A., Bzowej N.H., Chang K.M., Hwang J.P., Jonas M.M., Murad M.H. (2016). AASLD guidelines for treatment of chronic hepatitis B. Hepatology.

[20] European Association For The Study Of The Liver (2017). EASL 2017 Clinical Practice Guidelines on the management of hepatitis B virus infection. J. Hepatol..

[21] Iloeje U.H., Yang H.I., Su J., Jen C.L., You S.L., Chen C.J., The Risk Evaluation of Viral Load Elevation and Associated Liver Disease/Cancer-In HBV (the REVEAL-HBV) Study Group (2006). Predicting cirrhosis risk based on the level of circulating hepatitis B viral load. Gastroenterology.

[22] Li Y., Xie J., Han Y., Wang H., Zhu T., Wang N., Lv W., Guo F., Qiu Z., Du S. (2016). Lamivudine Monotherapy-Based cART Is Efficacious for HBV Treatment in HIV/HBV Coinfection When Baseline HBV DNA<20,000 IU/mL. J. Acquir. Immune Defic. Syndr..

[23] Idoko J., Meloni S., Muazu M., Nimzing L., Badung B., Hawkins C., Sankalé J.L., Ekong E., Murphy R., Kanki P. (2009). Impact of hepatitis B virus infection on human immunodeficiency virus response to antiretroviral therapy in Nigeria. Clin. Infect. Dis.

[24] Lok A.S., McMahon B.J. (2009). Chronic hepatitis B: Update 2009. Hepatology.

[25] Ive P., MacLeod W., Mkumla N., Orrell C., Jentsch U., Wallis C.L., Stevens W., Wood R., Sanne I., Bhattacharya D. (2013). Low prevalence of liver disease but regional differences in HBV treatment characteristics mark HIV/HBV co-infection in a South African HIV clinical trial. PloS ONE.

[26] Gu L., Han Y., Li Y., Zhu T., Song X., Huang Y., Yang F., Guan S., Xie J., Gohda J. (2015). Emergence of Lamivudine-Resistant HBV during Antiretroviral Therapy Including Lamivudine for Patients Coinfected with HIV and HBV in China. PloS ONE.

[27] Thio C.L., Smeaton L., Hollabaugh K., Saulynas M., Hwang H., Saravanan S., Kulkarni S., Hakim J., Nyirenda M., Iqbal H.S. (2015). Comparison of HBV-active HAART regimens in an HIV-HBV multinational cohort: Outcomes through 144 weeks. AIDS.

[28] Thio C.L., Smeaton L., Saulynas M., Hwang H., Saravanan S., Kulkarni S., Hakim J., Nyirenda M., Iqbal H.S., Lalloo U.G. (2013). Characterization of HIV-HBV coinfection in a multinational HIV-infected cohort. AIDS.

[29] Selabe S.G., Song E., Burnett R.J., Mphahlele M. (2009). Frequent detection of hepatitis B virus variants associated with lamivudine resistance in treated South African patients infected chronically with different HBV genotypes. J. Med. Virol..

[30] Kimbi G.C., Kramvis A., Kew M. (2004). Distinctive sequence characteristics of subgenotype A1 isolates of hepatitis B virus from South Africa. J. Gen. Virol..

[31] Amponsah-Dacosta E., Lebelo R.L., Rakgole J.N., Selabe S.G., Gededzha M.P., Mayaphi S.H., Powell E.A., Blackard J.T., Mphahlele M. (2015). Hepatitis B virus infection in post-vaccination South Africa: Occult HBV infection and circulating surface gene variants. J. Clin. Virol..

[32] Soriano V., McMahon B. (2013). Strategic use of lamivudine in the management of chronic hepatitis B. Antivir. Res..

[33] Soriano V., de Mendoza C., Peña J.M., Barreiro P. (2015). Advances in treating drug-resistant hepatitis B virus in HIV-infected patients. Expert Opin. Pharm..

